# The role of cognitive motivation and self-regulation in coping with occupational demands

**DOI:** 10.3389/fpsyg.2024.1422724

**Published:** 2024-12-04

**Authors:** Inga Hoff, Aniko Farkas, Ursula Melicherova, Volker Köllner, Jürgen Hoyer, Alexander Strobel, Anja Strobel

**Affiliations:** ^1^Personality Psychology and Assessment, Department of Psychology, Chemnitz University of Technology, Chemnitz, Germany; ^2^Psychosomatic Rehabilitation Research Group, Department of Psychosomatic Medicine, Charité – Universitätsmedizin Berlin, Berlin, Germany; ^3^Department of Psychosomatic Medicine, Rehabilitation Center Seehof, Federal German Pension Agency, Teltow, Germany; ^4^Behavioural Psychotherapy, Faculty of Psychology, Technische Universität Dresden, Dresden, Germany; ^5^Differential and Personality Psychology, Faculty of Psychology, Technische Universität Dresden, Dresden, Germany

**Keywords:** cognitive motivation, self-regulation, hardiness, mental health, occupational health

## Abstract

Cognitive motivation (COM) in conjunction with self-regulation (SR) was demonstrated to be positively linked to mental health in educational and clinical contexts. We introduce COM and SR in the occupational context and hypothesize these traits—based on their conceptual link to hardiness - to counteract health-risking stressors. Data from two large cross-sectional studies in Germany comprise a sample of 1,022 psychosomatic rehabilitation in-patients and a healthy sample of 298 employees of various occupations. Using bootstrapping in correlation analyses, we found in both samples COM and SR to be especially associated with an active, meaningful, and persistent coping style (0.31 ≤ *r* ≤ 0.57). Analyses via structural equation modeling found COM cross-sectionally predicting occupational coping via SR in both samples. We discuss COM and SR as personal resources with respect to Conservation of Resources Theory and provide ideas for promoting these variables in patients and employees.

## 1 Introduction

### 1.1 Mental health at the workplace

The issue of increasing exhaustion, psychological disorders, and resulting incapability to work has grown to be a severe problem for society. Data from 28 OECD countries indicated that 48% of people who were absent from work in the last year also experienced mental health problems (OECD, 2021). Moreover, 52% of European employees were at risk of depression in 2022 (OECD/European Union, 2022). In Germany, cases of sickness absence due to psychological disorders have increased 228% from 1997 to 2022 (DAK-Gesundheit, 2023). Although growing acceptance of psychological disorders and the accompanying willingness to also disclose such problems may be one of the reasons for the *immensely* increasing numbers, there is no denying the fact that a problem exists, and a solution should be found as soon as possible.

The factors surrounding this problem are manifold: organizations’ restructuring and the resulting uncertainty of occupation; increasing flexibility, mobility and adaptivity demands on employees; and an imbalance between engagement and occupational rewards have been established as risk factors for mental health (Siegrist, 2016). Further amplifying this issue, employees are currently facing challenges such as job threat in unknown dimensions due to the coronavirus pandemic and its consequences (e.g., Grandey et al., 2021). Reflecting underlying causes for increasing mental health problems such as capitalistic consumerism (e.g., Schaffner) and establishing policy interventions regarding the workplace are therefore essential (e.g., OECD, 2021). Nevertheless, the approach of the studies reported here was to examine the role of formerly neglected potential *personal* resources. We focus on self-regulative behavior that has often been linked to mental health (Tangney et al., 2004) and now will be widened by motivational aspects and cognitive investment (Von Stumm et al., 2011), which may offer further insight and advanced possibilities for intervention.

### 1.2 Self-regulative personality traits

Self-regulation (SR) is defined as “processes by which people control their thoughts, feelings, and behaviors” (Hoyle, 2006, p. 1507) in order to adjust behavior to goals, values, or other challenges in life. The corresponding personality trait dispositional self-control capacity (SC) refers to the ability to overcome or alter one’s reactions and to stop or resist unwanted behavioral tendencies (Tangney et al., 2004). SC has consistently been linked to positive health-related outcomes: better psychological adjustment, higher self-esteem and stronger interpersonal relationships (Tangney et al., 2004). SC is negatively associated with clinical symptoms, e.g., regarding alcohol-misuse, anxiety, and depression (Tangney et al., 2004) as well as with burnout (Duru et al., 2014). The individual fit regarding SC capacity and SC demands was found to be crucial for consequences of job strain (Schmidt et al., 2012).

Thus, a higher tendency to adjust behavior to the demands of the workplace has been established as an important personal resource. As for behavior adjustment, however, also attention needs to be shifted accordingly. Motivation as an important factor in initiating behavior can be considered in terms of shifting attention by determining the allocation of executive processing resources (Engelmann and Pessoa, 2014). Regarding interindividual differences, cognitive motivation (COM) (Enge et al., 2008) has been defined to be especially relevant for determining the allocation of cognitive resources. For a core variable of COM, Need for Cognition (NFC) (Cacioppo et al., 1996), associations with voluntary as well as automatic attention allocation were found (Enge et al., 2008). Defined as the tendency “to engage in and enjoy thinking” (Cacioppo and Petty, 1982, p. 116), NFC is typically associated with an inclination toward complexity (Cacioppo and Petty, 1982) and structure (Cohen et al., 1955). These observations resulted in a well-documented positive association with academic achievement, which is conceptualized by cognitive investment (Von Stumm and Ackerman, 2013; see section 1.3). In recent years, the view on NFC was expanded to its role regarding challenges in different areas of life: High-NFC individuals tend to deal with these challenges by active (Grass et al., 2018) and problem-focused coping, resulting in more positive affect (Bye and Pushkar, 2009). Individuals with higher NFC feel better (Bertrams and Dickhäuser, 2012; Fleischhauer et al., 2010) and show lower levels of neuroticism and symptoms of depression and anxiety (Epstein et al., 1996; Fleischhauer et al., 2019; Nishiguchi et al., 2016). Additionally, Strobel et al. (2021) assessed COM before in-patient’s treatment in psychosomatic rehabilitation and reported a negative association with depressive symptoms after treatment.

In conclusion, there is evidence that both COM and SR separately are related to successful coping with demanding life circumstances and thus more well-being and positive mental health outcomes. In this work, COM and SR will be used as umbrella terms whenever these aspects are addressed on a theoretical level. Specific study results, however, may refer to subordinate constructs like NFC for COM or SC for SR. In the following is outlined, how a potential combination of these traits can be conceptualized and may be relevant for occupational coping.

### 1.3 Cognitive motivation and self-regulation as a combined resource

The link between COM and SR has recently been examined: Grass et al. (2019) argue, that NFC’s main features should foster SC on an attentional as well as on a motivational level. A more elaborated way of processing information (which is typical for high NFC) is thought to make it easier to detect situations which require SC in the first place. Higher motivation to invest cognitive effort should support resisting unwanted behavior. First longitudinal data on the relationship between NFC and SC suggests, they may reciprocally strengthen each other over time (Nishiguchi et al., 2016). Being usually moderately associated, the relationship between NFC and SC could possibly be explained by individuals’ directedness toward goals (Kührt et al., 2021) and action (Grass et al., 2019). Recently it was shown that COM and SR can been subsumed into a second-order factor of *cognitive effort investment* (Kührt et al., 2021). Regarding different outcome variables, it could be shown that SC partially mediates the connection between NFC and school performance (Bertrams and Dickhäuser, 2009) and SC was found to mediate the association between NFC and affect-regulation (Bertrams and Dickhäuser, 2012). In a clinical setting, changes between admission and after treatment in COM partially mediated the association between changes in depressive symptoms and changes in SR (Strobel et al., 2021). Conceptually, the joint effect of COM and SR may be explained in terms of investment theory: COM was established as a trait, that enables individuals to spend resources in cognitive engagement and thus strengthen intellectual abilities in the process of seeking opportunities to learn and process experiences in a constructive way (Von Stumm and Ackerman, 2013). Thus, in this metanalysis COM was shown to be positively linked to crystalized intelligence and academic performance. As SR is associated with intellectual performance (e.g., Bertrams and Dickhäuser, 2009), investment in COM may also strengthen SR: COM may offer the requirements for initiating SR and therefore strengthen the ability to regulate oneself (Grass et al., 2018). SR in turn has been repeatedly shown to foster various coping behavior (Aldwin et al., 2011), while COM provides basic resources for behavior initiation in form of motivation, positive affect, and interests to foster a broad range of non-regulative traits and skills (see Strobel et al., 2017a,b).

These results indicate that COM and SR have a shared role in mental health, but also suggest a unique contribution of COM. Aspects supporting mental health which are specific for COM may include diverse interests, curiosity, and thrive for knowledge and finally the emotional aspect of COM as in the joy in cognitive endeavors (see Zerna et al., 2024). Also, COM’s unique contribution for psychological regulation may be explained *conceptually* by assuming a positive influence of higher COM levels on one’s hardiness (Kobasa, 1979). Being one of the most examined personal resources, the key features of hardiness are declared challenges (1), internal control beliefs (2), and commitment (3). These features can all be linked to NFC, and COM respectively: (1) Framing difficult situations as challenges is also typical for individuals high in NFC, e.g., in challenging situations in life (Strobel et al., 2017a). (2) Locus of control, the tendency to perceive circumstances as controllable, was found to be positively associated with NFC (Bye and Pushkar, 2009). (3) There is evidence for high-NFC individuals to have a higher commitment in terms of a deep involvement in different activities of life, which comes to effect in their consideration of others’ well-being in terms of moral behavior (Strobel et al., 2017b), higher levels of conscientiousness and persistence (Fleischhauer et al., 2010). Thus, COM can be considered a personal resource over and above SR.

Surprisingly, there is only little further research investigating COM as an occupational resource: NFC was found to be positively associated with job performance (Sojka and Deeter-Schmelz, 2008), and work motivation (Nowlin et al., 2018). Regarding well-being, there are a few studies linking NFC to job satisfaction, less exhaustion (Grass et al., 2023), positive affect at work, and psychological safety (see Zerna et al., 2024). In a teachers’ sample, SC was found to mediate the association between NFC and aspects of Burnout (Zerna et al., 2022). Additionally, Gallagher (2012) found NFC and the ability to manage resources to be negatively associated with a depressed mood at work.

Hence, to gain deeper insight into the management of challenges in work life, we sought to replicate central tenets of SR and expand them concerning a possible interplay with COM. Thus, we conducted two studies: Study 1 addressed COM, SR, and occupational coping in in-patients in psychosomatic rehabilitation and served as basis to form hypotheses. Study 2 addressed these traits, coping, and occupational outcomes in healthy employees to be able to extent the results of Study 1 to an area of possible application and intervention - the workplace.

## 2 Study 1

### 2.1 Hypotheses

Based on the aforementioned findings, and to specifically examine NFC as a resource at the workplace, in Study 1, we expected the following:

*H1*: COM and SR are positively related to occupational coping and negatively related to stress reactions, with COM being especially associated to an active and persistent coping style.

While Hypothesis 1 focuses on bivariate associations, in Hypothesis 2a we examine mediation effects, and additionally, in Hypothesis 2b to what extent COM has incremental validity over and above SR. Regarding recent mediation studies on COM and SR and conceptualizing COM as investment trait, we assume:

*H2a*: The positive association between COM and occupational coping and the negative association between COM and stress reactions are mediated by SR.

*H2b*: COM positively predicts occupational coping and negatively predicts stress reactions independent of SR.

### 2.2 Materials and methods

We report how we determined our sample size, all data exclusions (if any), all manipulations, and all measures in the study (Simmons et al., 2012). All data, analyzing scripts and materials for reproducing our analyses are permanently and openly accessible at https://osf.io/t457z/.

#### 2.2.1 Sample and data collection

This study was conducted in preparation of a multi-center clinical trial, which was approved by the Federal German Pension Agency (#8011–106-31/31.127). The sample consisted of as many adult in-patients who underwent psychosomatic rehabilitation between February 2018 and April 2019 as possible. The same sample was previously used for publication in Strobel et al. (2021). However, the analyses for this article are clearly distinguishable since other target variables are chosen and examined in context of COM and SR. Participating patients needed to be not suicidal or psychotic, be able to attend therapy (particularly group) sessions, and to have a good prognosis of regaining work ability (Köllner, 2016). During their clinic admission, patients were informed about the study and participants gave their written consent. The study was approved by the local ethics committee of Chemnitz University of Technology (V-250-15-AS-MOTIVATION-15012018). The data used in this study were collected under the supervision of a psychological technical assistant during the computer-based routine diagnostic procedure that each patient mandatorily completed before treatment. There was also a second assessment after treatment. Nonetheless, this was not analyzed in this study because the focus lies on occupational behavior, which cannot possibly differ from the first assessment for in-patients. A total of 1,060 patients participated in the study. The only instrument with missing data was the Occupational Stress and Coping Inventory. These cases were excluded. In the final sample (*N* = 1,022), 67.03% were female. Age varied between 19 and 64 years (*M* = 51.61, *SD* = 8.66), the majority was married (52.35%). Of all patients, 49.32% were declared unable to work, while 36.69% were not. The majority (59.98%) had vocational training listed as highest education level.

#### 2.2.2 Material

##### 2.2.2.1 Abridged cognitive effort scale

COM and SR were assessed with the *Abridged Cognitive Effort Scale* (ACES; Kührt et al., 2021; also see Strobel et al., 2021) which is a 24-item scale that assesses the individual tendency in cognitive effort. Two subscales (Need for Cognition, e.g., “I really enjoy a task that involves coming up with new solutions to problems.”), Intellect (e.g., “When I’m developing something new, I cannot rest until it’s completed.”) comprise the engagement in cognitively challenging tasks and situations (COM) and two further subscales (Self-Control, e.g., “I am able to work effectively toward long-term goals.”), Effortful Control (e.g., “Even when I feel energized, I can usually sit still without much trouble if it’s necessary.”) represent maintaining this engagement against inner and outer obstructions (effortful self-control, short ESC). Items are rated on a 7-point scale from *– 3 (strongly disagree)* to *+3 (strongly agree)*. Internal consistency of the scale was good (α = 0.72–0.85) except for the self-control scale (α = 0.57).

##### 2.2.2.2 Occupational stress and coping inventory (AVEM)

The AVEM (Eng. MECCA) (Schaarschmidt and Fischer, 2008) was used to assess occupational coping and stress reactions. It consists of 66 Items (e.g., “work is the most important purpose in my life.”) with 11 sub-dimensions (see Table 1). Participants were asked to indicate their agreement with the statements on a 5-point rating scale from *completely* to *not at all*. Additionally, there is an individual fit regarding four occupational behavior patterns, which are based on different weighing of the subscales’ values: *Type H* (healthy; successful coping and positive affection), *Type S* (unambitious, very low levels of commitment in combination with high emotional distance from work) *Type A* (tense, (too) high levels of effort and commitment combined with low coping abilities and emotional distancing), and finally, *Type B* (exhausted, resigned burnout-type). Internal consistency of all scales (α = 0.79–0.91) was good.

**Table 1:** Correlations between cognitive motivation, effortful self-control, and occupational coping in the clinical sample.

AVEM subscales and types	Cognitive motivation	Effortful self-control
Subjective significance of work	0.22*[0.13,0.29]	0.14*[0.05,0.22]
Career ambition	0.45*[0.38,0.51]	0.24*[0.16,0.32]
Commitment	0.14*[0.06,0.22]	0.05[−0.04,0.14]
Striving for perfection	0.12*[0.04,0.21]	0.08[−0.01,0.17]
Emotional distancing	0.13*[0.05,0.21]	0.20*[0.11,0.27]
Resignative tendencies	−0.33*[−0.40,-0.25]	−0.43*[−0.50,-0.37]
Active coping	0.57*[0.51,0.62]	0.53*[0.47,0.59]
Balance and mental stability	0.35*[0.28,0.42]	0.35*[0.27,0.43]
Satisfaction with work	0.36*[0.28,0.43]	0.41*[0.35,0.49]
Satisfaction with life	0.37*[0.29,0.45]	0.45*[0.38,0.51]
Experience of social support	0.17*[0.09,0.26]	0.29*[0.21,0.36]
Type S (unambitious)	0.12*[0.04,0.19]	0.22*[0.15,0.29]
Type H (healthy)	0.34*[0.29,0.40]	0.35*[0.29,0.41]
Type B (burnout)	−0.49*[−0.56,-0.43]	−0.49*[−0.55,-0.43]
Type A (tense)	0.32*[0.25,0.39]	0.24*[0.16,0.31]

Pearson correlation coefficients are displayed [0.99 Bootstrap confidence intervals]. *Confidence intervals do not include zero.

The complete data set includes a set of other constructs and demographic variables. Only those variables that are relevant for reproducing the present analyses are included in the data set accompanying this article, a full list of all variables is available.^1^

#### 2.2.3 Statistical analysis

All analyses were conducted with R Version 4.3.2 (R Core Team, 2023). For hypothesis H1, Pearson correlations and bootstrap-confidence-intervals (2000 repetitions) were calculated and evaluated as statistically significant if they did not include zero. Given that we tested H1 via 30 significance tests (see Table 1), we used adjusted confidence intervals of 1–0.05/30 = 0.998. Yet, because Bonferroni-correction may be too conservative in case of correlated measures, we chose a 99% CI for our analyses. With the final sample size of *N* = 1,022 participants, we were able to detect correlations of |*r*| ≥ 0.12 with a power of 1-β = 0.90 at a significance level of α’ = 0.01 as determined using the R package *pwr* (Champely, 2020). The size of these effects will be categorized normatively, following the guidelines by Gignac and Szodorai (2016) for individual difference research.

The assumption of linearity was tested graphically by a LOESS curve (span of 0.65). For H2, R package *lavaan* version 0.6–15 (Rosseel, 2012) was used to perform structural equation modeling. With our sample size of *N* = 1,022 and the degrees of freedom of our structural equation model of *df* = 16, we had a power of 1- β = 0.99 to detect misspecification of our model in terms of RMSEA = 0.06 at (*cf.* Hu and Bentler, 1999) α = 0.01 as determined using the R package *semPower* (Moshagen and Bader, 2023). Bootstrapping was applied on test statistics and standard errors. COM and ESC are based on two factors each (see materials section). Sum scores of these factors are used as indicators for the latent constructs as in Kührt et al. (2021). AVEM Types *H* and *B* were entered as manifest variables since they consist of only one indicator each. In the resulting model, all possible regression paths were tested using maximum likelihood estimation. The AVEM Types *S* and *A*, however, were not included in the model since our theory and conceptualization of both studies focuses on coping/health-promoting behavior and stress reactions. For completeness, we chose to use all AVEM types in the analyses of the bivariate associations.

### 2.3 Results

Descriptive statistics and intercorrelations between all scales and control variables can be found in Supplementary Appendix A.

#### 2.3.1 Hypothesis 1: associations

Correlation analysis revealed that COM and ESC were associated with coping with occupational demands (see Table 1). For all correlations except that of *commitment* and ESC and *striving for perfection* and ESC, the 99% CI did not include zero. Both COM and ESC were associated with the AVEM types. Especially strong were associations between *Type H (healthy-ambitious)* and COM and ESC and associations between *Type B (burnout-type)* and COM and ESC. The strongest associations of COM emerged with *active coping* and *career ambition* and the lowest with *emotional distancing* and *Type S (unambitious)*. Regarding ESC, the strongest associations could be observed for *active coping* and *satisfaction with life* and the lowest significant effects for *subjective significance of work* and *Type S*.

#### 2.3.2 Hypothesis 2a and 2b: mediated effects

**Figure 1:**
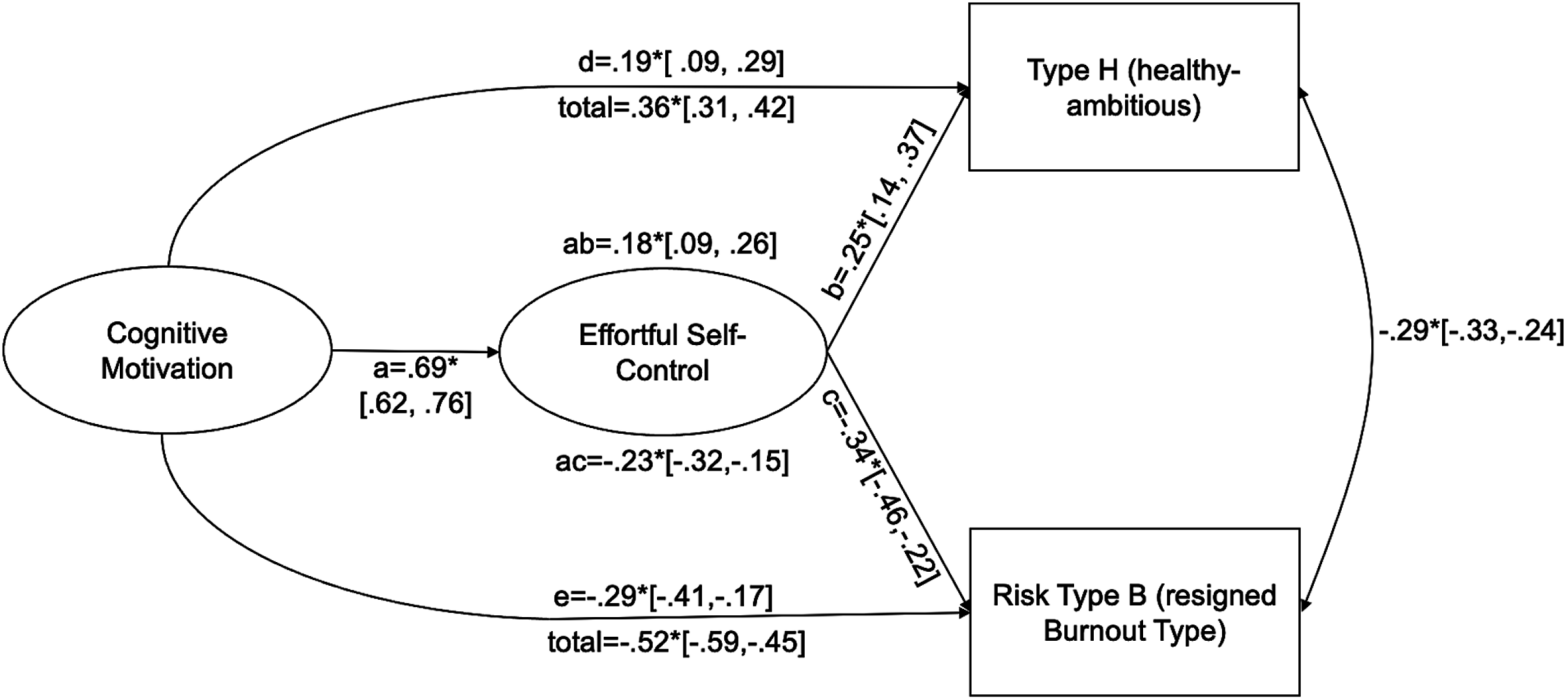
Cognitive Motivation predicting occupational coping via effortful self-control. Standardized regression/correlation coefficients and variances are displayed [0.99 bootstrap confidence intervals]. *Confidence intervals do not include zero.

For the structural equation model, the model-fit was acceptable (Bollen–Stine Adjusted Chi-Square [5] = 26.163, *p* = 0.001, CFI = 0.992, RMSEA = 0.064 [0.041, 0.090], SRMR = 0.015). In this model, COM and ESC cross-sectionally predicted *Types H* and *B* (Figure 1). COM strongly predicted ESC. There were small to medium-sized indirect effects of COM on *Type H* and on *Type B* via ESC. COM also directly predicted *Type H* and *Type B* (for further information on the model, see Supplementary Appendix B).

### 2.4 Summary

The aim of Study 1 was to investigate if and how COM and SR (in this study operationalized via ESC) can be considered personal resources for coping with occupational demands within a sample of in-patients in psychosomatic rehabilitation.

#### 2.4.1 Hypothesis 1: associations

Regarding the AVEM Types, COM and ESC were strongly negatively related to risk *Type B (burnout-type)*, while there were smaller, but also strong positive associations between these traits and *Type H* (healthy-ambitious). *Type B* is more strongly associated because especially the values of *Active coping* are stronger weighed in calculating *Type B* than for *Type H*. Since this subscale is strongly associated with COM and SR, the resulting correlations for *Type B* are expectable. COM and ESC were also weakly to moderately positively associated to AVEM *Types S (unambitious)* and *A* (tense). Again, these effects can be explained by the subscales’ different influences on the resulting AVEM-Types. Results for the subscales indicate that COM and ESC are related to health-promoting behavior. These positive aspects are to a certain degree also part of the *Types S and A* and explain why the self-regulative traits are positively associated with these less health promoting AVEM Types.

On the subscale level, strong associations were observed between COM, ESC, and *active coping* and *balance and mental stability* (positive) and *resignative tendencies* (negative). These especially large effects highlight particular aspects to be associated with these traits: Perceiving problems as challenges, inner calm, and persistence may be most relevant for understanding the link between occupational coping and COM and ESC. These traits were also linked to work ambition and satisfaction with work and life in general.

Associations between COM, ESC and *subjective significance of work, commitment, striving for perfection*, and *emotional distancing* and *experience of social support* were small compared to the other effects. These effects might be explained by the questions’ rather strong wording or specific limitations of the assessed constructs: For example, items for *significance of work* imply work being the only priority in one’s life and *commitment* does not refer to commitment in terms of hardiness but relates to overexertion at work. Both COM and ESC were expected to correlate positively with healthy levels of these constructs, which may explain the small associations found here. These small effects should be attempted to be replicated in further studies. If successful, these associations may be relevant in terms of practical significance, as even little explanation in behaviors such as overexertion may still matter immensely to the daily lives of those affected.

#### 2.4.2 Hypothesis 2a and 2b: mediated effects

In addition to the bivariate associations, a structural equation model focused on this work’s key aspects of health-promoting behavior and stress reactions. ESC partially mediated the association between COM occupational coping and stress reactions, supporting the idea of COM fostering SR (Grass et al., 2019). This also suggests these traits’ mutual role for coping with occupational demands and well-being at the workplace. Since this is a correlative design, this should be investigated in a longitudinal study, as causal statements cannot be made with this study. COM also predicted occupational coping and stress reactions directly, which highlights a unique contribution of COM over and above SR.

In conclusion, the results from the in-patients’ sample indicate that individuals high in COM as well as SR are far more likely to engage in health promoting behavior at work, while avoiding unhealthy behavior and reporting less stress reactions. This coping behavior especially includes an active, persistent, and mentally balanced approach toward difficulties at the workplace. Also, these individuals describe themselves as more ambitious and satisfied with life and work.

## 3 Study 2

### 3.1 Hypotheses

Given the results of Study 1, it needed to be investigated, if these findings also apply to healthy, currently working adults. Thus, COM and SR, and occupational coping were examined in Study 2 in such a sample. Expecting to find the results of Study 1 also in a healthy working sample, we assumed:

*H1*: COM and SR are positively related to occupational coping, including a more active, persistent, and meaningful coping style as well as more satisfaction with life and career, while being negatively related to stress reactions.

*H2a*: The positive association between COM and occupational coping and the negative association between COM and stress reactions are mediated by SR.

*H2b*: COM positively predicts occupational coping and negatively predicts stress reactions independent of SR.

Given the considerations for COM and job satisfaction and findings on job performance (Grass et al., 2023; Sojka and Deeter-Schmelz, 2008) together with the assumption, that successful coping with occupational demands results in positive occupational outcomes, we added job satisfaction and job performance as outcome variables to our hypothesis:

*H2c*: The positive associations between COM and job satisfaction and job performance are mediated by SR, occupational coping, and stress reactions.

### 3.2 Materials and methods

All data, analyzing scripts and materials for reproducing our analyses are permanently and openly accessible (see text footnote 1). We report how we determined our sample size, all data exclusions (if any), all manipulations, and all measures in the study (Simmons et al., 2012). The analyses were not pre-registered.

#### 3.2.1 Sample and data collection

The procedure was evaluated and approved by the Ethics Committee of Chemnitz University of Technology (V-348-15-IH-Arbeitsleben-30072019). It was not considered to require further ethical approvals and hence, as uncritical concerning ethical aspects according to the criteria used by the Ethics Committee including aspects of the sample of healthy adults, voluntary attendance, noninvasive measures, no deception, and appropriate physical and mental demands on the subject. A sample size of 250 was aimed for because according to Schönbrodt and Perugini (2013), stabile estimation of correlation coefficients (for small to medium-sized *r*) in differential psychology requires a sample size of at least 250. A total of 299 individuals from Germany, Austria and Switzerland were recruited in September 2019 via SoSci Panel (see Leiner, 2017), meeting the inclusion criteria of being currently employed (at least 50% of full time) and having a minimum working experience of 2 years. All participants provided written informed consent. One individual was excluded due to completion time of about only 4 minutes. In the final sample of *N* = 298, 57.0% described their gender as female and 0.3% as diverse. Age ranged between 21 and 68 (*M* = 44.32, *SD* = 11.30) and the level of education was rather high with 87.57% being academics. Working hours per week including overtime ranged between 19 and 66 (*M* = 39.58, *SD* = 7.94), and there was a variety of occupational sectors, including increased numbers in health system and social welfare. Participants were offered additional information about occupational coping in general and to enter a prize draw for 50€ twice per 100 participants.

#### 3.2.2 Materials

##### 3.2.2.1 Abridged cognitive effort scale

As in Study 1, COM and SR were measured with the Abridged Cognitive Effort Scale (ACES; see Study 1). Internal consistency of the scale was good (α = 0.68–0.78).

##### 3.2.2.2 Scale for assessment of resilient behavior in the workplace

Differing from Study 1, occupational coping was assessed by the 16-item scale for assessment of resilient behavior in the workplace (Soucek et al., 2015) due to accessibility. The four subscales *emotional coping* (e.g., “I know how to calm myself, when I am under a lot of pressure at work.”), *comprehensive planning*, *positive reframing*, and *focused action* each represent specific aspects of a persistent, active, and optimistic coping style with occupational demands. Internal consistency of the scale was good (α = 0.79–0.91).

##### 3.2.2.3 Stress and coping inventory

Stress reactions were assessed with the subscale *stress symptoms* of the SCI (Satow, 2012). It includes 13 Items (e.g., “I often brood over my life”). Internal consistency of the scale was good (α = 0.84).

##### 3.2.2.4 Career satisfaction scale

This five item-scale (Abele et al., 2011) was used to assess sense of achievement (e.g., “I am satisfied with the progress in my general previous career.”). Internal consistency of the scale was good (α = 0.87).

##### 3.2.2.5 Job performance

Self-reported job performance was assessed by a German translation of two items by Robinson (1996).

##### 3.2.2.6 Job satisfaction

This was measured by a one item smiley-scale from the FIDES-project (Reineboth et al., 2018).

##### 3.2.2.7 Satisfaction with life scale

Satisfaction with life was measured by a scale of the same title (Janke and Glöckner-Rist, 2014). It comprises five items (e.g., “In most areas, my life fulfills my ideal.”) Internal consistency of the scale was very good (α = 0.89).

Additionally, demographic variables were included. Further variables were assessed but were excluded for this analysis due to no correspondence in the clinical sample. A full list of all variables is available (see text footnote 1).

#### 3.2.3 Statistical analysis

All analyses were carried out in the same way as in Study 1. With our sample size of *N* = 298 participants, we were able to detect correlations of |*r*| ≥ 0.17 with a power of 1-β = 0.65 at a significance level of α’ = 0.01. Regarding the structural equation model, with our sample size of *N* = 298 and the degrees of freedom of *df* = 91, we had a power of 1- β = 0.99 to detect misspecification of our model in terms of RMSEA = 0.06 at α = 0.01. To correspond to AVEM type *H* in Study 1, a primary factor was assumed for the RBW scale, which is supported by a confirmatory factor analysis. For RBW, four parcels were constructed by dividing the items equally by subscales (Matsunaga, 2008). For Stress Symptoms, three items with factor loadings of at least 0.7 were selected. Problematic negative residual variances for the indicator *intellect* were resolved by fixating this parameter to zero, which is in line with the factor loading and estimation of variance (see Supplementary Appendix D).

### 3.3 Results

Descriptive statistics and intercorrelations for all scales and control variables can be found in Supplementary Appendix C.

#### 3.3.1 Hypothesis 1: associations

Correlation analysis revealed that COM and ESC were associated with occupational coping (see Table 2). For all correlations, the 99% CI did not include zero. *Resilient behavior in the workplace* was strongly positively associated with both COM and ESC. *Stress symptoms* were moderately negatively associated with COM and strongly negatively associated with ESC. Beyond that, the strongest association of COM emerged with *positive reframing* and *focused* action and the lowest with life satisfaction and career satisfaction. Regarding ESC, the strongest associations could be observed for focused action and comprehensive planning and the lowest significant effects for life satisfaction and positive reframing.

**Table 2:** Correlations between cognitive motivation, effortful self-control, and occupational coping in the healthy sample.

	Cognitive motivation	Effortful self-control
Emotional coping	0.26*[0.11,0.40]	0.35*[0.21,0.49]
Comprehensive planning	0.40*[0.23,0.52]	0.39*[0.23,0.52]
Positive reframing	0.46*[0.32,0.56]	0.31*[0.15,0.44]
Focused action	0.43*[0.31,0.56]	0.49*[0.32,0.61]
Resilient behavior in the workplace	0.49*[0.35,0.60]	0.48*[0.29,0.61]
Stress symptoms	−0.20*[−0.33, −0.05]	−0.36*[−0.48, −0.22]
Life satisfaction	0.20*[0.03,0.37]	0.34*[0.20,0.48]
Career satisfaction	0.17*[0.01,0.33]	0.38*[0.24,0.50]

Pearson correlation coefficients are displayed [0.99 Bootstrap confidence intervals]. *Confidence intervals do not include zero.

#### 3.3.2 Hypothesis 2a, 2b, and 2c: mediated effects

**Figure 2:**
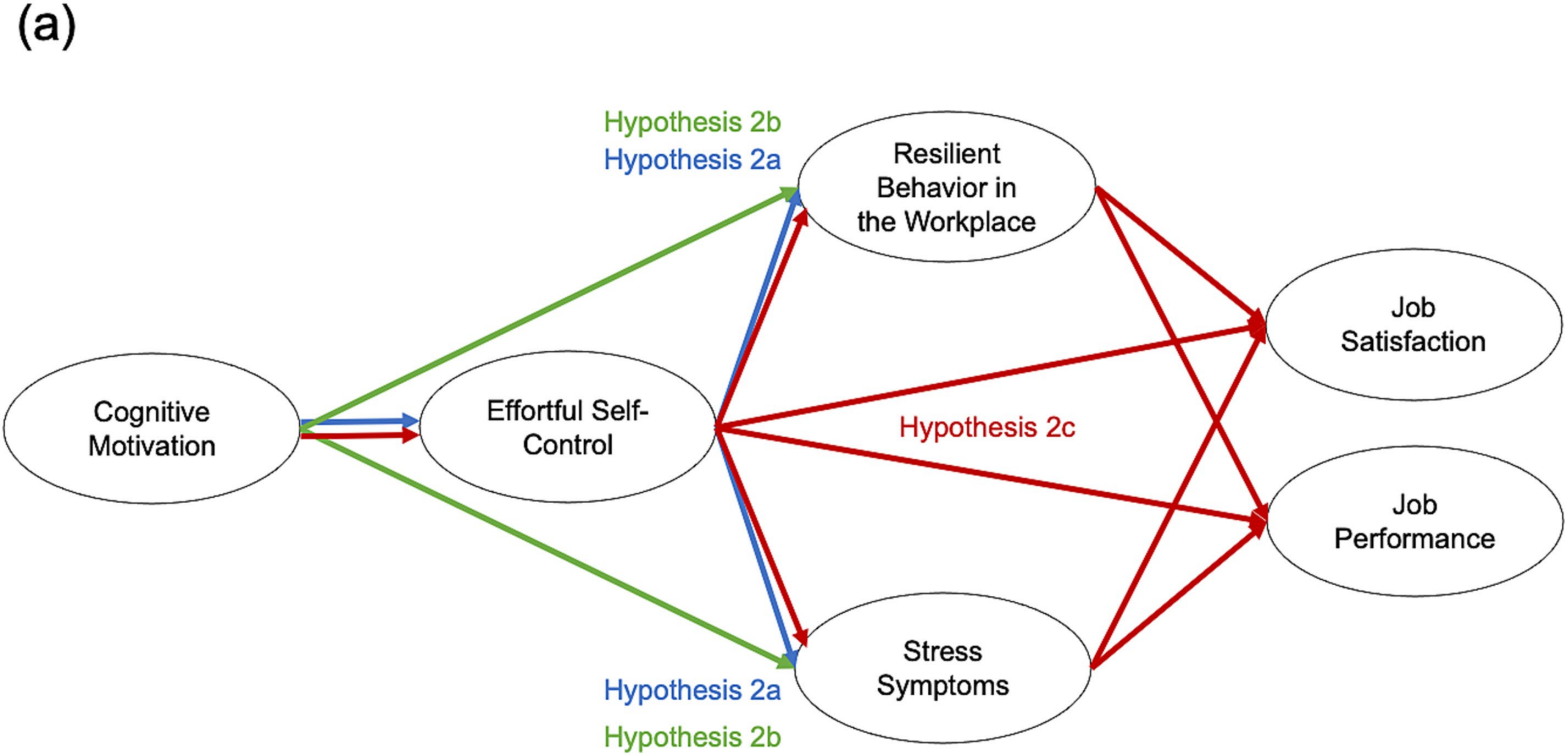
Cognitive motivation predicting Occupational Outcomes via effortful self-control and occupational coping. **(A)** Hypotheses color-coded in model. **(B)** Full model with color-coded effects. Standardized regression/correlation coefficients are displayed [0.99 bootstrap confidence intervals]. Effects <0.1 omitted for clarity. Statistically significant indirect effects: ab = 0.21*[0.06, 0.36], ac = −0.23*[−0.36, −0.09], am = 0.25*[0.10, 0.39], acf = 0.07*[0.02, 0.13], cf = 0.15*[0.04, 0.25], hd = 0.08*[0.01, 0.15]. *Confidence intervals do not include zero.

For the structural equation model, the model-fit was acceptable (Bollen–Stine Adjusted Chi-Square [64] = 179.527, *p* < 0.001, CFI = 0.953, RMSEA = 0.078 [0.064, 0.091], SRMR = 0.044). In this model, COM and ESC predicted *resilient behavior at the workplace (RBW)*, *stress symptoms* and occupational outcomes cross-sectionally (Figure 2). COM strongly predicted ESC. There were medium-sized indirect effects of COM on *RBW* and on *stress symptoms* via ESC. COM also directly predicted RBW and ESC predicted *RBW* and *stress symptoms*. There were also very small indirect effects of COM on job satisfaction via *RBW* and via ESC and *stress symptoms*. ESC mediated the association between COM and job performance, which was also strongly predicted by ESC. *Stress symptoms* strongly predicted job satisfaction (for further information on the model, see Supplementary Appendix E).

### 3.4 Summary

The aim of this study was to investigate if and how COM and SR (in this study operationalized via ESC) can be considered personal resources for occupational coping. To this end, a sample of healthy employees was analyzed.

#### 3.4.1 Hypothesis 1: associations

COM and ESC were positively associated with occupational coping and well-being, particular aspects were *comprehensive planning, emotional coping*, life and career satisfaction. Negative associations were observed between COM, ESC, and stress reactions. The positive effects regarding *focused action* and *positive reframing* were especially strong and thus highlight the importance of these aspects for coping with demands at the workplace.

#### 3.4.2 Hypothesis 2a, 2b, and 2c: indirect effects

ESC mediated the associations between COM and occupational coping as well as stress reactions. This indicates COM fostering ESC, which supports employees’ coping with occupational demands and well-being. Due to the cross-sectional design, this is a statistical and no causal statement. COM also predicted occupational coping and job satisfaction via occupational coping independent of ESC, which highlights its unique contribution and may stress motivational factors being important beyond the path via ESC. Apart from ESC, COM and stress reactions were unrelated, which suggests COM *per se* being more relevant in terms of positive and health-promoting aspects. Job performance was strongly predicted by ESC. This is plausible since there are certain aspects of ESC, which are crucial to job performance, such as starting unattractive tasks or stopping counterproductive work-behavior (De Boer et al., 2015). But still, there was an indirect effect of COM on job performance via ESC. Thus, also in terms of job satisfaction, the shared role of COM and ESC becomes apparent. This is also reflected in the indirect effect of COM on job satisfaction via ESC and stress reactions.

Overall, Study 2 implicates that individuals high in COM and ESC were much more likely to engage in health-promoting behavior at the workplace, especially dealing with demands in an active, optimistic, and persistent way. They also report higher satisfaction with work and higher job performance more often.

## 4 Discussion

The issue of employees’ mental health was approached by focusing on the self-regulative personality traits COM and SR. Results from a sample of in-patients in psychosomatic rehabilitation (Study 1) and a sample of healthy employees (Study 2) showed that COM and SR were strongly associated with occupational coping and negatively related to stress reactions. A particularly important aspect was an active, meaningful, and persistent approach toward difficulties at the workplace. The assumption of COM supporting SR and thus enabling occupational coping was supported on a cross-sectional level. Additionally in Study 2, there were mediation effects of these traits on job performance and job satisfaction via stress reactions. COM showed a unique contribution over and above SR regarding coping and job satisfaction. In line with our findings, Kührt et al. (2021) observed different correlation patterns for COM and SR regarding other constructs such as the BIG-5 dimensions openness, conscientiousness, and neuroticism. They also demonstrated that assessing COM and SR as *one* higher-order construct is possible and may be beneficial in research on demand avoidance when focusing on these traits’ shared variance. Zerna et al. (2024) discussed both constructs in terms of well-being: Both NFC and SC may share a desire for control, while NFC offers its unique aspect of positive emotionality.

While in both studies associations with occupational coping were strong, stress reactions were only weakly associated with COM and SR in Study 2, and COM did not directly predict stress reactions independent of SR. This can be explained by the differing operationalizations: In Study 1, behavioral tendencies at coping with occupational demands were used, whereas in Study 2 concrete indicators for stress including physical and mental symptoms were considered. The self-regulative traits should relate more strongly to behavioral tendencies than concrete physical and mental body reactions. Also, the effects of Study 2 are in line with findings on depressed mood at work (Gallagher, 2012). Besides, recent findings on COM, SR, and depressive symptoms in a clinical setting (Strobel et al., 2021) are in line with the results for Study 1, suggesting higher variance and thus higher effects for in-patients than for the general population. Since recent effects regarding NFC as a core aspect of COM and burnout (e.g., Fleischhauer et al., 2019; Schmidt et al., 2012) are considerably smaller, the discovered effects of COM, SR and health-promoting/endangering behavior are quite astonishing and can be seen as a first confirmation for the notion of these traits being an important personal resource in work contexts.

### 4.1 Active, meaningful, and persistent coping

There are several specific aspects of coping, which are similar in both studies: The effects regarding *balance* and *inner stability* (Study 1) and *emotional coping* (Study 2) are quite similar since they both refer to calming oneself at work. They can be compared in terms of effect sizes (not direction of the effect) to previous findings on NFC and neuroticism (e.g., Fleischhauer et al., 2019; Sevincer et al., 2017), as the individual tendency to be emotionally unstable. Also, previous work by Strobel et al. (2017a) on NFC and positive emotionality yielded similar results to this study. Besides, the most relevant aspects of occupational coping in terms of COM and SR in both studies were also key aspects of hardiness: Firstly, persistence and a positive future perspective when facing difficulties and/or failure is an important feature of COM (Fleischhauer et al., 2010) and SR by definition can also be linked to commitment in terms of hardiness. Secondly, framing problems at work as challenges and opportunities to grow was also an important aspect in both samples and directly related to hardiness’ feature challenges. Effects in this study are stronger compared to previous results on NFC and coping (Bye and Pushkar, 2009; Grass et al., 2018), which may also highlight the added value of the hardiness-related coping aspects when examining COM and SR. We conclude that these aspects are important for understanding successful coping with occupational demands in general. Therefore, these results contribute to a better understanding of occupational coping, COM, and SR as resources, and stress the role of hardiness as a theoretical link. Furthermore, the results support the assumption on a cross-sectional level, that COM is being invested in SR and that this process fosters health-promoting behavior at the workplace. The results underline COM being an investment trait and expand this theory regarding SR in the occupational context: The aspect of processing experiences in a constructive way to foster cognitive growth and learning (see Von Stumm and Ackerman, 2013) was reflected by COM being strongly associated with the tendency to frame occupational difficulties as challenges and opportunities to grow. Additionally, *comprehensive planning* (Study 2) can be discussed in terms of one of COM’s main aspects since it involves a structured approach toward problems. Thus, in this study it could be shown, that this aspect is also reflected in the occupational context. Small positive associations between COM, SR and *experience of social support* (Study 1) could be found, which is in line with findings by Tanas et al. (2020) and stresses COM and SR as resources also in terms of social variables.

### 4.2 Cognitive motivation, self-regulation, and occupational outcomes

Considering the associated occupational outcomes, effects for job and life satisfaction were stronger in Study 1. The results of Study 2 expand upon comparable research on NFC and motivation to work (Nowlin et al., 2018) and NFC and life satisfaction (Yazdani and Siedlecki, 2021) in healthy adults. Regarding job performance, the results are in line with the work of Sojka and Deeter-Schmelz (2008) and are also comparable to effects in student samples (Grass et al., 2018). Concerning satisfaction with life and work, stronger variance and thus stronger effects for in-patients are expectable.

Overall, even though relatively small, the effects between COM, SR and work-related outcomes are meaningful, considering relating non-context-specific personality traits to behavior in a specific context (Gignac and Szodorai, 2016). The associations between COM, SR, and occupational coping can be described as similar in the samples of in-patients and healthy employees. Above their role for specific coping behavior, these traits can also be thought of as positive influences on mental health at the workplace, since the assessed coping behavior is linked to various indicators of mental and physical well-being (Schaarschmidt and Fischer, 2008; Soucek et al., 2015). The effect sizes are surprising, since abstract personality and behavior in a specific context are linked, especially considering this was not controlled for features of the organization. Because the participants in both samples were in very different life circumstances and mental states, it is very remarkable that the findings are comparable, which stresses a general important role of COM and SR when coping with occupational demands. Both studies support on a cross-sectional level the idea of COM being invested in SR, which in turn is invested in health-promoting behavior at the workplace. However, as Zerna et al. (2022) pointed out in terms of Burnout, COM may exceed its health-promoting influence on occupational coping in highly health-risking work conditions. Individuals high in COM may for example overestimate their resources in such scenarios and therefore may be especially inclined to Burnout-promoting behavior. Future research should therefore investigate COM and SR in context of specific work conditions and explore limits of personal resources in very high-demanding settings. Also, as highlighted earlier, changes in work conditions on an organizational as well as a political level are essential and cannot be replaced by promoting personal resources.

All in all, these studies highlight the shared important role of COM and SR as well as COM’s unique contribution as resources in the occupational context in both in-patients and healthy employees. This resource supports coping with occupational demands in a way, that it helps people stay perseverant on tasks and perceive problems as meaningful challenges. This and other aspects of an active, optimistic approach toward problems at work characterize a health-promoting behavior at the workplace, which individuals high in COM and SR tend to choose. Thus, these individuals report less stress reactions and are more satisfied with life and work.

### 4.3 Limitations

As a limiting factor for the interpretation of these results, the decreased reliability of the ESC subscale *self-control* in Study 1 can be seen. The results, however, are in line with the previous SC literature. Moreover, this issue did not occur in Study 2, so in any case conclusions from this sample are assumed to be conclusive. Further limitations concern the cross-sectional design, especially regarding the interplay of COM and SR, and lack of additional organizational variables. The specific influence of educational levels, occupational sectors, and work conditions on personal resources such as COM and SR was not investigated in this study and should be addressed in future research. Although the instruments corresponding to the clinical sample were carefully chosen, it should be noted that coping behavior in both samples was operationalized differently. Thus, further investigation should include a longitudinal study design so that causal effects between COM and SR, as well as additional variables concerning the organization, general work and life conditions and further organizational attitudes can be investigated.

## 5 Conclusion and implications

In conclusion, this work presents firsts evidence for COM, that is the tendency and joy to engage in cognitive tasks, and SR, the ability to control one’s own cognitions, emotions, and behaviors toward a goal, being highly relevant personal resources in coping with everyday stressors and demands of working life. On a conceptual level, this study provides a better understanding of the link between COM and SR and the relevance of hardiness in this context. Since COM has been argued to be an important resource initiating a broad range of these positive outcomes, promoting COM in employees could be a valuable addition to management of occupational health. Possible options for this include specific training programs, communicating this trait to be beneficial to the organization, and working conditions that do not suppress, but enable the joy of thinking. Since COM is a personality trait comprising all areas of life, these results are not restricted to academic jobs. In occupations that are naturally high-demanding, COM and SR may be also relevant for personnel selection. These prospects have strong potential to be an important element in addressing the issue of employees’ severely increasing exhaustion and inability to work.

## Data Availability

The datasets presented in this study can be found in online repositories. The names of the repository/repositories and accession number(s) can be found at: Open Science Forum repository (https://osf.io/t457z/).

## Footnotes

^1^ https://osf.io/t457z/
