# Supplementary material for: The role of cognitive motivation and self-regulation in coping with occupational demands
Source: Front Psychol. 2024 Dec 4;15:1422724. doi: 10.3389/fpsyg.2024.1422724 (PMC11652205; doi:10.3389/fpsyg.2024.1422724)
Supplement: Supplementary file 1 [file Data_Sheet_1.PDF]

Supplementary Material

1 Appendix A

	1	2	3	4	5	6	7	8	9	10	11	12	13	14	15	16	17	18	19	20	21	22	Mean	SD	Range
Subjective significance of work (1)	(.88)	.50*	.35*	.23*	-.16*	.02	.31*	.05	.21*	.12*	-.04	-.24*	.09	-.44*	.69*	.21*	.20*	.09	.15*	.22*	.14*	.20*	15.73	5.05	6.00 - 30.00
Career ambition (2)	.50*	(.82)	.34*	.27*	-.11	.01	.42*	.10	.27*	.15*	-.04	-.24*	.33*	-.43*	.55*	.42*	.42*	.16*	.27*	.45*	.24*	.40*	15.52	4.69	3.00 - 30.00
Commitment (3)	.35*	.34*	(.85)	.55*	-.50*	.21*	.24*	-.16*	.23*	-.05	-.14*	-.44*	.05	-.16*	.52*	.10	.16*	-.01	.09	.14*	.05	.11	20.77	5.00	6.00 - 30.00
Striving for perfection (4)	.23*	.27*	.55*	(.86)	-.38*	.38*	.24*	-.15*	.14*	-.06	-.03	-.42*	.08	-.07	.38*	.05	.18*	.04	.10	.12*	.08	.12*	23.49	4.33	6.00 - 30.00
Emotional distancing (5)	-.16*	-.11*	-.50*	-.38*	(.91)	-.48*	.13*	.35*	.06	.30*	.20*	.59*	.24*	-.25*	-.30*	.14*	.11	.15*	.20*	.13*	.20*	.18*	15.20	5.33	6.00 - 30.00
Resignative tendencies (6)	.02	.01	.21*	.38*	-.48*	(.87)	-.43*	-.46*	-.36*	-.44*	-.21*	-.54*	-.34*	.52*	-.01	-.36*	-.27*	-.37*	-.42*	-.33*	-.43*	-.42*	19.61	4.77	6.00 - 30.00
Active coping (7)	.31*	.42*	.24*	.24*	.13*	-.43*	(.80)	.33*	.51*	.50*	.23*	.13*	.43*	-.63*	.44*	.51*	.57*	.40*	.55*	.57*	.53*	.62*	18.73	4.03	6.00 - 30.00
Balance and mental stability (8)	.05	.10*	-.16*	-.15*	.35*	-.46*	.33*	(.79)	.30*	.35*	.20*	.41*	.33*	-.44*	.03	.34*	.32*	.29*	.35*	.35*	.35*	.40*	16.66	4.32	6.00 - 29.00
Satisfaction with work (9)	.21*	.27*	.23*	.14*	.06	-.36*	.51*	.30*	(.89)	.59*	.30*	.20*	.37*	-.62*	.39*	.32*	.35*	.34*	.41*	.36*	.41*	.43*	20.03	4.74	6.00 - 30.00
Satisfaction with life (10)	.12*	.15*	-.05	-.06	.30*	-.44*	.50*	.35*	.59*	(.85)	.44*	.35*	.44*	-.60*	.21*	.34*	.36*	.38*	.43*	.37*	.45*	.46*	17.24	4.64	6.00 - 30.00
Experience of social support (11)	-.04	-.04	-.14*	-.03	.20*	-.21*	.23*	.20*	.30*	.44*	(.79)	.27*	.30*	-.29*	-.03	.15*	.18*	.27*	.25*	.17*	.29*	.25*	21.07	4.37	6.00 - 30.00
Type S - unambitious (12)	-.24*	-.24*	-.44*	-.42*	.59*	-.54*	.13*	.41*	.20*	.35*	.27*	(.00)	.04	-.44*	-.30*	.14*	.08	.20*	.20*	.12*	.22*	.18*	12.60	26.43	0.00 - 100.00
Type H - healthy (13)	.09*	.33*	.05	.08*	.24*	-.34*	.43*	.33*	.37*	.44*	.30*	.04	(.00)	-.45*	-.01	.33*	.32*	.28*	.35*	.34*	.35*	.39*	6.00	17.18	0.00 - 100.00

Risk Type B - burnout (14)	-.44*	-.43*	-.16*	-.07	-.25*	.52*	-.63*	-.44*	-.62*	-.60*	-.29*	-.44*	-.45*	(.00)	-.62*	-.47*	-.45*	-.39*	-.49*	-.49*	-.49*	-.56*	56.48	39.51	0.00 - 100.00
Type A - tense (15)	.69*	.55*	.52*	.38*	-.30*	-.01	.44*	.03	.39*	.21*	-.03	-.30*	-.01	-.62*	(.00)	.29*	.32*	.17*	.25*	.32*	.24*	.32*	24.92	32.59	0.00 - 100.00
Need for Cognition (16)	.21*	.42*	.10*	.05	.14*	-.36*	.51*	.34*	.32*	.34*	.15*	.14*	.33*	-.47*	.29*	(.77)	.77*	.39*	.53*	.94*	.51*	.84*	0.29	7.62	-18.00 - 18.00
Intellect (17)	.20*	.42*	.16*	.18*	.11*	-.27*	.57*	.32*	.35*	.36*	.18*	.08*	.32*	-.45*	.32*	.77*	(.85)	.40*	.60*	.94*	.56*	.87*	2.99	8.13	-18.00 - 18.00
Self-Control (18)	.09*	.16*	-.01	.04	.15*	-.37*	.40*	.29*	.34*	.38*	.27*	.20*	.28*	-.39*	.17*	.39*	.40*	(.57)	.63*	.42*	.89*	.71*	-0.36	6.17	-18.00 - 18.00
Effortful Control (19)	.15*	.27*	.09*	.10*	.20*	-.42*	.55*	.35*	.41*	.43*	.25*	.20*	.35*	-.49*	.25*	.53*	.60*	.63*	(.72)	.60*	.91*	.83*	1.24	6.85	-18.00 - 18.00
Cognitive Motivation (20)	.22*	.45*	.14*	.12*	.13*	-.33*	.57*	.35*	.36*	.37*	.17*	.12*	.34*	-.49*	.32*	.94*	.94*	.42*	.60*	(.89)	.57*	.91*	3.28	14.81	-36.00 - 36.00
Effortful Self-Control (21)	.14*	.24*	.05	.08*	.20*	-.43*	.53*	.35*	.41*	.45*	.29*	.22*	.35*	-.49*	.24*	.51*	.56*	.89*	.91*	.57*	(.79)	.86*	0.88	11.74	-34.00 - 32.00
Cognitive Effort Investment (22)	.20*	.40*	.11*	.12*	.18*	-.42*	.62*	.40*	.43*	.46*	.25*	.18*	.39*	-.56*	.32*	.84*	.87*	.71*	.83*	.91*	.86*	(.79)	4.16	23.57	-68.00 - 65.00

Appendix A. Intercorrelations between all variables, Means, and Standard-Deviations in Study 1 (clinical sample). Pearson correlation coefficients, means, standard deviations, and range of scores are displayed. Internal consistencies (Cronbach’s alpha) are presented in the diagonal. *$p < 0.01$ (Bonferroni-Holm-adjusted above the diagonal).

Indicator Variables	Factor loadings
Intellect	.91[.87, .95]
Need for Cognition	.84[.81, .88]
Self-Control Scale	.67[.61, .73]
Effortful Control Scale	.93[.88, .98]
Latent Variables	Regression coefficients
Effortful Self-Control ~ Cognitive Motivation (a)	.69[.62, .76]
AVEM Burnout ~ Effortful Self-Control (b)	.25[.14, .37]
AVEM Burnout ~ Cognitive Motivation (e)	-.29[-.41, -.17]
AVEM Healthy ~ Cognitive Motivation (d)	.19[.09, .29]
AVEM Healthy ~ Effortful Self-Control (b)	.25[.14, .37]
Latent Variables	Covariances
AVEM burnout ~ AVEM Healthy	.25[.14, .37]
Variables	Variances
Intellect	.18[.11, .25]
Need for Cognition	.29[.22, .35]
Self-Control Scale	.55[.47, .63]
Effortful Control Scale	.13[.03, .23]
AVEM Burnout	.67[.60, .74]
AVEM Healthy	.83[.79, .88]
Cognitive Motivation	1[1, 1]
Effortful Self-Control	.52[.43, .61]
Indirect and total effects	Regression coefficients
axb	.18[.09, .26]
axc	-.23[-.32, -.15]
total_H	.36[.31, .42]
total_B	-.52[-.59, -.45]

Appendix B. Factor loadings, regression coefficients, covariances, and variances for the structural equation model in Study 1 (clinical sample). Standardized coefficients [.99 Bootstrap Confidence Intervals] are displayed.

3 Appendix C

	1	2	3	4	5	6	7	8	9	10	11	12	13	14	15	16	17	Mean	SD	Range
Need for Cognition (1)	(.73)	.65*	.32*	.30*	.92*	.34*	.74*	.21*	.27*	.41*	.36*	.40*	-.19	.18	.25*	.17	.18	6.62	5.78	-13.00 - 18.00
Intellect (2)	.65*	(.78)	.34*	.50*	.89*	.44*	.78*	.26*	.47*	.42*	.43*	.50*	-.17	.19	.19	.14	.07	9.18	4.92	-13.00 - 18.00
Self-Control (3)	.32*	.34*	(.74)	.69*	.36*	.93*	.78*	.33*	.38*	.25*	.41*	.43*	-.36*	.34*	.40*	.40*	.24*	3.20	6.16	-14.00 - 18.00
Effortful Control (4)	.30*	.50*	.69*	(.68)	.43*	.90*	.80*	.32*	.34*	.33*	.49*	.46*	-.30*	.29*	.33*	.30*	.21*	6.03	5.11	-10.00 - 18.00
Cognitive Motivation (5)	.92*	.89*	.36*	.43*	(.84)	.43*	.83*	.26*	.40*	.46*	.43*	.49*	-.20*	.20*	.25*	.17	.14	15.81	9.72	-26.00 - 36.00
Effortful Self-Control (6)	.34*	.44*	.93*	.90*	.43*	(.83)	.86*	.35*	.39*	.31*	.49*	.48*	-.36*	.34*	.40*	.38*	.25*	9.23	10.36	-20.00 - 36.00
Cognitive Effort	.74*	.78*	.78*	.80*	.83*	.86*	(.87)	.36*	.47*	.45*	.54*	.57*	-.33*	.32*	.39*	.33*	.23*	25.04	16.97	-43.00 - 65.00
Emotional coping (8)	.21*	.26*	.33*	.32*	.26*	.35*	.36*	(.90)	.45*	.50*	.50*	.78*	-.40*	.31*	.30*	.36*	.35*	5.06	1.27	1.00 - 7.00
Comprehensive planning	.27*	.47*	.38*	.34*	.40*	.39*	.47*	.45*	(.86)	.41*	.48*	.73*	-.18	.19	.23*	.15	.13	5.05	1.18	1.00 - 7.00
Positive reframing (10)	.41*	.42*	.25*	.33*	.46*	.31*	.45*	.50*	.41*	(.91)	.64*	.82*	-.32*	.36*	.26*	.35*	.42*	5.00	1.38	1.00 - 7.00
Focused action (11)	.36*	.43*	.41*	.49*	.43*	.49*	.54*	.50*	.48*	.64*	(.79)	.82*	-.36*	.23*	.34*	.31*	.22*	4.74	1.14	1.00 - 7.00
Resilient behavior at the	.40*	.50*	.43*	.46*	.49*	.48*	.57*	.78*	.73*	.82*	.82*	(.92)	-.40*	.35*	.36*	.38*	.36*	4.96	0.98	1.00 - 7.00
Stress symptoms (13)	-.19*	-.17*	-.36*	-.30*	-.20*	-.36*	-.33*	-.40*	-.18*	-.32*	-.36*	-.40*	(.84)	-.40*	-.21*	-.57*	-.48*	1.78	0.53	1.00 - 3.46
Career satisfaction (14)	.18*	.19*	.34*	.29*	.20*	.34*	.32*	.31*	.19*	.36*	.23*	.35*	-.40*	(.87)	.39*	.65*	.58*	3.74	0.82	1.40 - 5.00
Job performance (15)	.25*	.19*	.40*	.33*	.25*	.40*	.39*	.30*	.23*	.26*	.34*	.36*	-.21*	.39*	(.00)	.38*	.40*	4.23	0.62	1.50 - 5.00
Life satisfaction (17)	.17*	.14	.40*	.30*	.17*	.38*	.33*	.36*	.15*	.35*	.31*	.38*	-.57*	.65*	.38*	(.89)	.56*	5.03	1.19	1.40 - 7.00
Job satisfaction (18)	.18*	.07	.24*	.21*	.14	.25*	.23*	.35*	.13	.42*	.22*	.36*	-.48*	.58*	.40*	.56*	(.00)	5.16	1.24	1.00 - 7.00

Appendix C. Intercorrelations between all variables, Means, and Standard-Deviations in Study 2 (healthy sample). Pearson correlation coefficients, means, standard deviations, and range of scores are displayed. Internal consistencies (Cronbach's alpha) are presented in the diagonal. *$p < 0.01$ (Bonferroni-Holm-adjusted above the diagonal).

Appendix D

Indicator Variables	Factor loadings
Need for Cognition	.69[.54, .83]
Intellect	.95[.78, 1.12]
Self-Control Scale	.81[.70, .92]
Effortful Control Scale	.86[.74, .97]
Resilient behavior at the workplace Parcel1	.85[.79, .91]
Resilient behavior at the workplace Parcel2	.91[.87, .95]
Resilient behavior at the workplace Parcel3	.93[.90, .95]
Resilient behavior at the workplace Parcel4	.93[.90, .96]
Stress symptoms item 5	.75[.67, .82]
Stress symptoms item 6	.88[.80, .96]
Stress symptoms item 7	.70[.59, .80]
Job satisfaction item	1[1, 1]
Job performance item 1	.75[.57, .93]
Job performance item 2	.64[.49, .80]
**Latent Variables**	**Regression Coefficients**
Resilient behavior at the workplace ~ Cognitive Motivation (h)	.33[.10, .57]
Stress symptoms ~ Cognitive Motivation (i)	-.01[-.22, .21]
Job satisfaction ~ Cognitive Motivation (j)	-.16[-.38, .05]
Job performance ~ Cognitive Motivation (k)	-.12[-.40, .16]
Job satisfaction ~ Effortful Self-Control (l)	.06[-.21, .34]
Job performance ~ Effortful Self-Control (m)	.48[.20, .76]
Effortful Self-Control ~ Cognitive Motivation (a)	.54[.35, .73]
Resilient behavior at the workplace ~ Effortful Self-Control (b)	.38[.14, .62]
Stress symptoms ~ Effortful Self-Control (c)	-.43[-.65, -.22]
Job satisfaction ~ resilient behavior at the workplace (d)	.26[.03, .50]
Job performance ~ resilient behavior at the workplace (e)	.22[-.05, .49]
Job satisfaction ~ stress symptoms (f)	-.33[-.52, -.15]
Job performance ~ stress symptoms (g)	-.04[-.26, .18]
**Latent Variables**	**Covariances**
Resilient behavior at the workplace, stress symptoms	-.31[-.50, -.13]
Job satisfaction, job performance	.38[.16, .60]

Variables	Variances
Need for Cognition	.53[.33, .73]
Intellect	.10[-.23, .43]
Self-Control Scale	.35[.17, .53]
Effortful Control Scale	.27[.07, .47]
Resilient behavior at the workplace Parcel1	.28[.18, .37]
Resilient behavior at the workplace Parcel2	.17[.10, .24]
Resilient behavior at the workplace Parcel3	.14[.09, .19]
Resilient behavior at the workplace Parcel4	.13[.07, .18]
Stress symptoms item 5	.44[.33, .56]
Stress symptoms item 6	.23[.09, .37]
Stress symptoms item 7	.51[.37, .66]
Job satisfaction item	0[0, 0]
Job performance item 1	.44[.17, .71]
Job performance item 2	.58[.38, .79]
Cognitive Motivation	1[1, 1]
Effortful Self-Control	.71[.51, .91]
Resilient behavior at the workplace	.60[.45, .76]
Stress symptoms	.81[.66, .96]
Job satisfaction	.75[.63, .88]
Job performance	.65[.45, .86]
**Indirect and total effects**	**Regression coefficients**
axb	.21[.05, .36]
axc	-.23[-.38,-.09]
axl	.03[-.11, .18]
axm	.26[.09, .43]
axbxd	.05[-.02, .13]
axbxe	.05[-.02, .11]
axcxf	.08[.01, .14]
axcxg	.01[-.04, .06]
bxd	.10[-.03, .23]
bxe	.08[-.04, .20]
cxf	.14[.03, .26]

cxg	.02[-.08, .11]
hxd	.09[.00, .17]
hxe	.07[-.02, .17]
ixf	.00[-.07, .07]
ixg	.00[-.01, .01]
Total job satisfaction	.31[.02, .60]
Total job performance	.34[.02, .67]

Appendix D. Factor loadings, regression coefficients, covariances, and variances for the structural equation model in Study 2 (healthy sample; first approach). Standardized coefficients [.99 Bootstrap Confidence Intervals] are displayed.

For the structural equation model in Study 2, 440 of 2,000 bootstrap draws were returned by the function as non-admissible solutions. Further examination of these cases revealed that all of them showed negative residual variances for the indicator *intellect*. These caused the respective models to not converge. Considering the very high factor loading of the indicator ($\lambda = .95$), it is plausible to assume that there is no residual variance, which is also supported by the statistical parameters concerning the residual variance ($\varepsilon = 2.41$, $SE = 2.96$, $p = .415$). Thus, the residual variance for the indicator *intellect* will be fixated to zero.

5 Appendix E

Indicator Variables	Factor loadings
Need for Cognition	.65[.56, .74]
Intellect	1[1, 1]
Self-Control Scale	.80[.71, .89]
Effortful Control Scale	.86[.77, .96]
Resilient behavior at the workplace Parcel1	.85[.80, .90]
Resilient behavior at the workplace Parcel2	.91[.87, .95]
Resilient behavior at the workplace Parcel3	.93[.90, .95]
Resilient behavior at the workplace Parcel4	.93[.90, .96]
Stress symptoms item 5	.75[.67, .83]
Stress symptoms item 6	.88[.80, .95]
Stress symptoms item 7	.70[.59, .80]
Job satisfaction item	1[1, 1]
Job performance item 1	.75[.58, .91]
Job performance item 2	.65[.51, .79]
**Latent Variables**	**Regression Coefficients**
Resilient behavior at the workplace ~ Cognitive Motivation (h)	.30[.08, .51]
Stress symptoms ~ Cognitive Motivation (i)	.01[-.18, .19]
Job satisfaction ~ Cognitive Motivation (j)	-.16[-.36, .04]
Job performance ~ Cognitive Motivation (k)	-.13[-.36, .10]
Job satisfaction ~ Effortful Self-Control (l)	.06[-.21, .33]
Job performance ~ Effortful Self-Control (m)	.48[.24, .72]
Effortful Self-Control ~ Cognitive Motivation (a)	.52[.35, .68]
Resilient behavior at the workplace ~ Effortful Self-Control (b)	.41[.18, .64]
Stress symptoms ~ Effortful Self-Control (c)	-.44[-.63,-.24]
Job satisfaction ~ resilient behavior at the workplace (d)	.26[.04, .48]
Job performance ~ resilient behavior at the workplace (e)	.22[-.02, .46]
Job satisfaction ~ stress symptoms (f)	-.33[-.51, -.15]
Job performance ~ stress symptoms (g)	-.04[-.25, .17]
**Latent Variables**	**Covariances**
Resilient behavior at the workplace, stress symptoms	-.32[-.49, -.14]

Job satisfaction, job performance	.37[.18, .57]
Variables	Variances
Need for Cognition	.58[.46, .69]
Intellect	.00[.00, .00]
Self-Control Scale	.36[.21, .50]
Effortful Control Scale	.26[.10, .42]
Resilient behavior at the workplace Parcel1	.28[.19, .37]
Resilient behavior at the workplace Parcel2	.17[.10, .24]
Resilient behavior at the workplace Parcel3	.14[.09, .19]
Resilient behavior at the workplace Parcel4	.13[.07, .18]
Stress symptoms item 5	.44[.33, .56]
Stress symptoms item 6	.23[.10, .36]
Stress symptoms item 7	.51[.37, .66]
Job satisfaction item	.00[.00, .00]
Job performance item 1	.44[.19, .69]
Job performance item 2	.58[.40, .76]
Cognitive Motivation	1[1, 1]
Effortful Self-Control	.73[.56, .91]
Resilient behavior at the workplace	.62[.47, .76]
Stress symptoms	.81[.67, .95]
Job satisfaction	.75[.63, .87]
Job performance	.66[.47, .84]
Indirect and total effects	Regression coefficients
axb	.21[.06, .36]
axc	-.23[-.36,-.09]
axl	.03[-.11, .17]
axm	.25[.10, .39]
axbxd	.05[-.02, .13]
axbxe	.05[-.02, .11]
axcxf	.07[.02, .13]
axcxg	.01[-.04, .06]
bxd	.11[-.02, .23]
bxe	.09[-.02, .20]

cxf	.15[.04, .25]
cxg	.02[-.08, .11]
hxd	.08[.01, .15]
hxe	.07[-.02, .15]
ixf	.00[-.06, .06]
ixg	.00[-.01, .01]
Total job satisfaction	.31[.03, .59]
Total job performance	.33[.04, .63]

Appendix E. Factor loadings, regression coefficients, covariances, and variances for the structural equation model in Study 2 (healthy sample; final model). Standardized coefficients [.99 Bootstrap Confidence Intervals] are displayed.
